# An amphiphilic-ligand-modified gold nanoflower probe for enhancing the stability of lateral flow immunoassays in dried distillers grains†

**DOI:** 10.1039/c9ra06690j

**Published:** 2019-11-11

**Authors:** Tongtong Ma, Hong Duan, Wenjing Zhang, Yanna Shao, Liangwen Hao, Xirui Chen, Yuankui Leng, Xiaolin Huang, Yonghua Xiong

**Affiliations:** State Key Laboratory of Food Science and Technology, Nanchang University Nanchang 330047 P. R. China yuankuilengxq@163.com yhxiongchen@163.com; Jiangxi-OAI Joint Research Institute, Nanchang University Nanchang 330047 P. R. China; School of Food Science and Technology, Nanchang University Nanchang 330047 P. R. China; Jiangxi Key Laboratory for Microscale Interdisciplinary Study P. R. China

## Abstract

An amphiphilic ligand-capped gold nanoflower (AuNF) was proposed as a novel lateral flow immunoassay (LFA) reporter for zearalenone (ZEN) detection in distillers dried grains solubles (DDGS). The amphiphilic ligand consists of a thiol-terminated hydrophobic alkane chain, a tetra (ethylene glycol) unit, and a terminal carboxyl group. The novel AuNF probe (N-AuNF-Abs) was prepared by coupling the amino group of anti-ZEN antibodies with the AuNF carboxyl group *via* an amido covalent linkage. For comparison, a traditional AuNF probe (Tr-AuNF-Abs) was prepared by labeling antibodies on the surface of citrate capped AuNFs *via* an electrostatic adsorption method. The detection performance of the two probes in LFA was systematically investigated, including the half maximal inhibitory concentration (IC_50_), robustness and reproducibility for ZEN quantitative detection in DDGS samples, and shelf life. The N-AuNF-Ab based LFA (N-LFA) had a lower IC_50_ value (15.97 ng mL^−1^) for ZEN detection in phosphate buffered saline than that of the Tr-AuNF-mAb based LFA (Tr-LFA, 31.06 ng mL^−1^). The IC_50_ value of N-LFA in DDGS extract was 17.46 ng mL^−1^, whereas the Tr-LFA showed poor robustness and reproducibility in DDGS samples, resulting in a failed determination. The intra- and inter-assays of N-LFA for ZEN-spiked DDGS samples indicated that the average recoveries ranged from 93.0% to 125.9%, with coefficients of variation ranging from 2.8% to 21.9%. These results indicated that the N-LFA strip exhibits good robustness and an acceptable accuracy for ZEN quantitative detection in complex DDGS samples. In accelerated aging studies, N-LFA showed a longer shelf life (5 years) than that of Tr-LFA (1 year). In summary, the proposed method provided a novel strategy to prepare a super-stable probe for enhancing the detection performance of LFA for small molecular detection in complex sample matrices such as DDGS.

## Abstract 2

An amphiphilic ligand-capped gold nanoflower (AuNF) was proposed as a novel lateral flow immunoassay (LFA) reporter for zearalenone (ZEN) detection in distillers dried grains solubles (DDGS).

## Graphical abstract

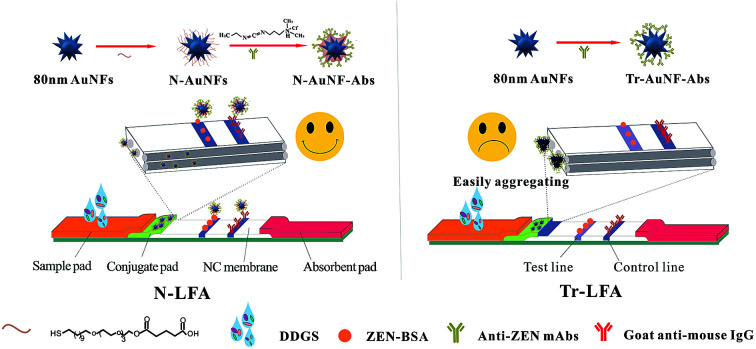

## 1. Introduction

Lateral flow immunoassays (LFAs) are powerful tools for on-site screening detection in clinical diagnosis, environmental analysis, and food safety monitoring because of their simplicity, rapid results, cost-effectiveness, and user-friendliness.^1–5^ Currently, various nanomaterials, including light absorbing nanoparticles^6–8^ (*e.g.*, gold nanoparticles (AuNPs), carbon nanoparticles, and colloidal selenium), light emitting nanoparticles^9–13^ (*e.g.*, dye-doped fluorescent beads, time-resolved fluorescent beads, quantum dot beads, and up-converting phosphor nanoparticles), and magnetic nanoparticles,^14^ have been widely used as labels in LFAs. Among them, AuNPs are the most popular signal reporter in commercial LFAs because of their facile synthesis and modification, biocompatibility and low toxicity, low cost, and naked eye detectable results.^15,16^ The AuNPs traditionally used in LFAs are usually prepared *via* the trisodium citrate reduction method, in which trisodium citrate acts as both a reductant and a stabilizer during the AuNP synthesis.^17^ The resultant AuNPs typically have a core–shell structure, in which the atomic Au aggregate that forms the core is surrounded by an electrostatic double layer, and the citrate ligands impart a negative charge onto the surface of the AuNPs.^18,19^ These citrate-capped AuNPs are sensitive to the electrolyte, which can shrink the electrostatic double layers on the AuNPs and cause AuNP agglutination. Various macromolecules, such as water-soluble proteins, single-stranded DNA, carbohydrates, and polymers can adsorb onto the surface of the AuNPs through dative bonds and *via* electrostatic and hydrophobic forces.^20^ Therefore, the classical coupling protocol for antibody-labeled AuNPs (referred to from hereon as traditional AuNP and antibody conjugates, Tr-AuNP-Abs) consists of simply adding the antibodies (Abs) to the AuNP solution at a certain pH value.^21^ To enhance the stability of the Tr-AuNP-Abs and reduce the nonspecific binding of Tr-AuNP-Abs on the nitrocellulose (NC) membrane, blocking agents, such as proteins (*e.g.*, bovine serum albumin), sugar (*e.g.*, sucrose and mannitose), polymers (*e.g.*, poly(ethylene glycol), PEG), are required to block the excess binding sites on the AuNPs.^22–24^ However, owing to the weak binding affinity between antibodies and AuNPs, the stability of Tr-AuNP-Abs remains a serious problem when they are applied to complex sample matrices containing high levels of water-soluble proteins, metal ions, and complex carbohydrates. Therefore, using Tr-AuNP-Abs based LFAs (Tr-LFAs) for this kind of sample matrix often leads to poor reproducibility and even failed detection.

**Scheme 1:**
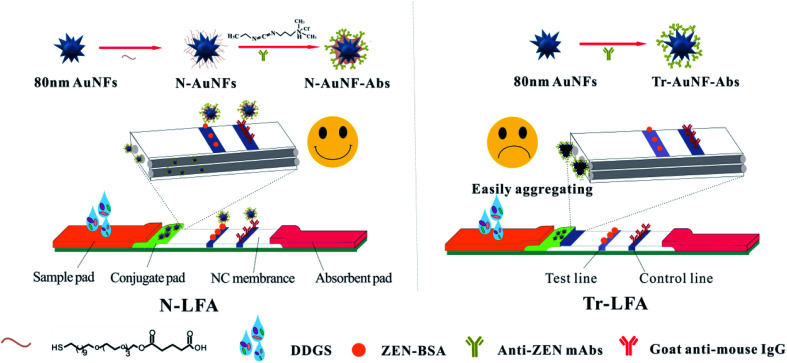
Schematics of the strips based on N-AuNF-Abs and Tr-AuNF-Abs for the detection of ZEN in DDGS.

In the present study, an amphiphilic ligand (Scheme 1) was used to replace the citrate group on the AuNPs with strong Au–S bonds.^25^ The amphiphilic ligand consists of a hydrophobic alkane chain, a tetra (ethylene glycol) unit, and a terminal carboxyl group. The alkane chains self-assembled into a hydrophobic layer on the surface of the AuNPs, which can prevent the ligands from detaching from the surface of the AuNPs in the presence of high concentrations of chemical compounds or complex bio-macromolecules, because it is impossible for these water-soluble compounds to penetrate the hydrophobic layer and reach the surface of the AuNPs. The tetra (ethylene glycol) unit was chosen to improve the solubility because many research groups have reported that PEG-decorated AuNPs show better biocompatibility and solubility than citrate-capped AuNPs in most sample solutions.^26,27^ Furthermore, the terminal carboxyl groups of the proposed ligand were used to conjugate the antibodies for the target analyte *via* covalent bonds. Compared with conventional spherical AuNPs, multi-branched gold nanoflowers (AuNFs) exhibit stronger localized surface plasmon resonance (LSPR) absorption, better colloidal stability, and higher antibody loading capacity due to their rough surfaces and large surface-to-volume ratios.^28–30^ Our preliminary work demonstrated that better LFA detection performance was obtained using AuNFs than spherical AuNPs in the same format.^25,31,32^

Dried distillers grain with solubles (DDGS) is a byproduct of the alcoholic fermentation of cereal grains for beverages or fuel.^33^ DDGS has been recognized as a valuable foodstuff that provides energy, water-soluble proteins, vitamins, and minerals for animals.^34^ However, DDGS obtained from corn can potentially contain a higher concentration of zearalenone (ZEN) than was present in the corn prior to fermentation.^35^ ZEN is an estrogenic and carcinogenic mycotoxin produced by *Fusarium* species, in particular, *F. graminearum*, *F. culmorum*, and *F. crookwellense*. Corn is easily contaminated by *Fusarium* species in many parts of the world. The ingestion of ZEN by animals may cause early sexual maturity and promote the occurrence of DNA adducts and micronuclei by triggering chromosome aberrations.^36,37^

In this work, AuNFs modified with an amphiphilic ligand were used to conjugate anti-ZEN monoclonal antibodies *via* covalent coupling of the amino group of Abs to the carboxyl group of AuNF by a carbodiimide method. The proposed AuNF probe was used as a novel probe (N-AuNF-Abs) of LFA (N-LFA) for the quantitative detection of ZEN in DDGS samples. The detection performance of N-LFA in terms of its resistance to matrix interference, sensitivity and accuracy, and storage stability were investigated and compared with those of Tr-LFA. The results indicated that N-AuNF-Abs exhibited superior performance as a probe for the LFA detection of small molecular pollutants in the complex sample matrix.

## 2. Model and methods

### 2.1. Materials and instruments

*N*-(3-(Dimethylamino) propyl)-*N*′-ethylcarbodiimidehydrochloride (EDC·HCl), zearalenone (ZEN), and bovine serum albumin (BSA) were purchased from Sigma-Aldrich Chemical (St. Louis, MO). ZEN–BSA conjugates (mole ratio of 15 : 1) and anti-ZEN mAbs were prepared in our laboratory. Donkey anti-mouse IgG antibodies were purchased from Beijing Zhongshan Biotechnology, Inc. (Beijing, China). The sample pad, nitrocellulose (NC) membrane, and absorbent pad were obtained from Schleicher and Schuell GmbH (Dassel, Germany). The ZEN free DDGS samples (determined by liquid chromatography-tandem mass spectrometry, LC-MS/MS) were collected from different local farms. The phosphate-buffered saline (PBS, 0.01 M, pH = 7.4) was obtained by adding 1.22 g of K_2_HPO_4_, 1.36 g of KH_2_PO_4_, and 8.5 g of NaCl in 1000 mL of Milli-Q water and adjusted to 7.4, unless otherwise specified, before use. Phosphate buffer (PB; 0.01 M, pH 7.0) was prepared by mixing stock solutions of NaH_2_PO_4_ and Na_2_HPO_4_. All of the other reagents were of analytical grade or better and purchased from Sinopharm Chemical Corp. (Shanghai, China).

The BioDot XYZ platform combined with a motion controller, BioJet Quanti 3000k dispenser and AirJet Quanti 3000k dispenser for solution dispensing was supplied by BioDot (Irvine, CA, USA).

### 2.2. Preparation of citrate-stabilized AuNFs and amphiphilic-ligand-decorated AuNFs

Citrate-capped AuNFs (Tr-AuNFs) with an average diameter of 80 nm were synthesized using a gold seed-mediated growth approach according to a previous report with slight modifications.^38,39^ Firstly, 2.7 mL of a 1% sodium citrate solution was added to 100 mL of a boiling 0.01% gold chloride trihydrate solution under constant stirring. 18 nm gold seeds were obtained after 10 min of reaction. 1.78 mL of the above gold seed-containing solution was added to 100 mL of ultrapure water under vigorous stirring. Once the temperature reached 55 °C, 1.2 mL of a 1% HAuCl_4_ solution, 2.64 mL of a 1% sodium citrate solution, and 24 mL of 0.03 M hydroquinone solution were immediately added in sequence. The 80 nm Tr-AuNFs were obtained after another 10 min of heating. The concentration of the resulting Tr-AuNFs was calculated based on the dosage of 18 nm seeds.

The amphiphilic ligand-decorated AuNFs (N-AuNFs) were obtained *via* exchanging the citrate group of the Tr-AuNFs with an amphiphilic ligand.^40,41^ The Tr-AuNFs were mixed with an excess of the amphiphilic ligand in weakly alkaline water (pH = 9.0) at ambient temperature for 4 h under magnetic stirring. The N-AuNFs were purified *via* centrifugation and re-suspended in ultrapure water. The Tr-AuNFs and N-AuNFs were stored at 4 °C for further use.

### 2.3. Preparation of the two kinds of AuNF probes

N-AuNF-Abs was synthesized by coupling the amino group of anti-ZEN mAbs with the carboxyl group on the surface of N-AuNFs *via* an amide bond.^25^ Typically, 0.3 pmol of N-AuNFs was first mixed with 37.5 μg of unpurified ascites containing 4% anti-ZEN mAbs in 15 mL of a 0.01 M phosphate buffer (PB, pH = 6.0) at ambient temperature for 30 min under magnetic stirring. Three portions of a solution containing 75 μg EDC were added to the solution individually, each addition was followed by 30 min of reaction to form amide bonds. 500 μL of 10% BSA (w/v) was then added to the above mixture, and another two portions of EDC solution (75 μg) were added separately, followed by 30 min of reaction each. The as-prepared N-AuNF-Abs was then purified *via* centrifugation and re-suspended in 0.01 M PB buffer (pH = 7.4) containing 25% sucrose and 0.1% sodium azide.

Tr-AuNF-Abs was prepared by immobilizing anti-ZEN mAbs on the surface of the Tr-AuNFs *via* electrostatic adsorption.^31,32^ Typically, 18.9 μg of unpurified ascites was added to 15 mL of Tr-AuNF solution (20 pM) with a pH of 7.9. After 1 h of incubation under gentle stirring at 25 °C, 500 μL of 10% BSA (w/v) was added, and the solution was incubated for another 60 min. The as-prepared Tr-AuNF-Abs was then purified *via* centrifugation and resuspended in 0.01 M PB buffer (pH = 7.4) containing 25% sucrose and 0.1% sodium azide. Both types of AuNF probes were stored at 4 °C until further use.

### 2.4. Fabrication of the ZEN-LFA

The LFA comprised four parts: a sample pad, conjugate pad, nitrocellulose (NC) membrane, and absorbent pad. The sample pad was soaked in 0.1 M PBS (pH = 8.0) solution containing 1.0% (w/v) BSA, 0.1% (w/v) sodium azide, and 0.25% (w/v) Tween-20, and then dried at 60 °C for 2 h. The conjugate pad (glass fiber membrane) was doused with 0.01 M PBS (pH = 7.4) containing 0.2% Tween-20 and 2% sucrose and then dried at 60 °C for 4 h. Both kinds of synthetic AuNF probes were uniformly sprayed onto separate conjugate pads using a gold spraying instrument and then vacuum-dried at 37 °C for 90 min. Donkey anti-mouse IgG and ZEN–BSA conjugate were spotted on the NC membrane with a density of 0.74 μL cm^−1^ as the control (C) and test (T) lines, respectively, and then dried at 37 °C for 12 h. The pretreated sample pad, conjugate pad, NC membrane, and absorption pad were assembled and then cut into 3.9 mm × 60 mm strips. All the LFA strips were packaged in a plastic bag containing a desiccant gel and stored at ambient temperature prior to use. The typical detection procedure was as follows: 70 μL of sample solution was added to the well of the sample pad. After 15 min of incubation, the optical densities of the T (OD_T_) and C (OD_C_) lines were recorded using an optical strip reader (Shanghai Huguo Science Instrument Co., Ltd., Shanghai, China).

## 3. Results and discussion

### 3.1. Preparation and characterization of the AuNFs

The Tr-AuNFs were synthesized *via* a gold seed-mediated growth method using hydroquinone as the reductant. The morphology and size distribution of the Tr-AuNFs were characterized using a high-resolution transmission electron microscope (TEM, JEOL JEM 2100, Tokyo, Japan). The resultant AuNFs exhibited a flower-like morphology with spikes and good monodispersity with an average size of 80 nm (Fig. S1A†). Dynamic light scattering (DLS) analysis indicated that the average hydrodynamic diameter (*D*_H_) of the Tr-AuNFs was about 90 nm (Fig. S1C†). In the UV-vis spectrum in Fig. S1D,† the most intense surface plasmon resonance (SPR) peak of the Tr-AuNFs was located at 630 nm. To improve the robustness of LFA, N-AuNFs, which had enhanced stability and high resistance against matrix interference, were obtained by replacing the citrate group on the surface of the Tr-AuNFs with an amphiphilic ligand *via* a ligand exchange process (Scheme 1). The TEM image (Fig. S1B†), average *D*_H_ (Fig. S1C†) and SPR peak (Fig. S1D†) of the resultant N-AuNFs were nearly the same as those of the Tr-AuNFs.

**Fig. 1:**
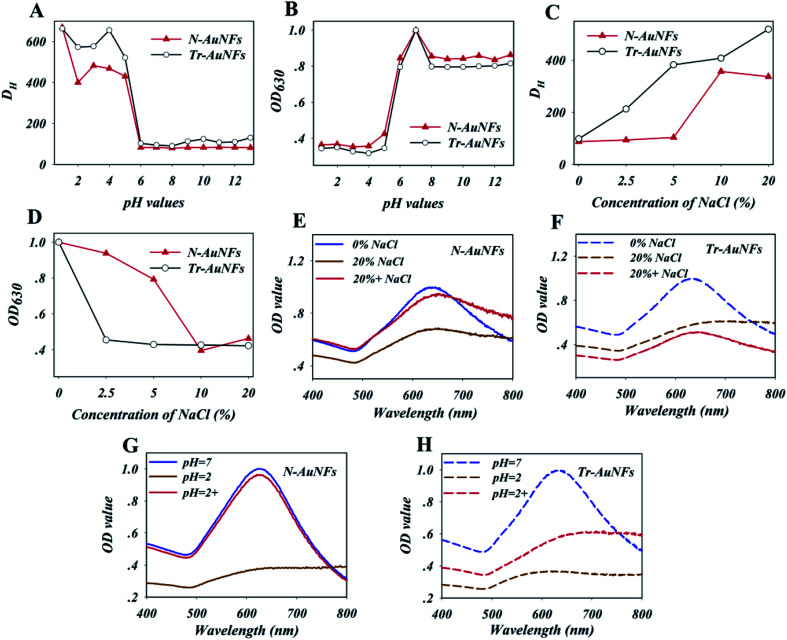
The effects of the pH values and NaCl concentrations on the hydration diameters (A and C) and optical intensity (OD_630_ value, B and D) of the N-AuNF and Tr-AuNF solutions, respectively. UV-Vis spectra of N-AuNF (E) and Tr-AuNF (F) treated with and without 20% of NaCl solution (wt%); UV-Vis spectra of N-AuNF (G) and Tr-AuNF (H) with a strongly acidic solution (pH = 2); N-AuNF 0% NaCl and Tr-AuNF 0% NaCl represent that N-AuNFs and Tr-AuNFs dissolve in pure water; N-AuNF 20% NaCl and Tr-AuNF 20% NaCl represent that N-AuNFs and Tr-AuNFs dissolve in 20% of NaCl solution (wt%); N-AuNFs 20% + NaCl and Tr-AuNFs 20% + NaCl represent that the aggregated N-AuNFs and aggregated Tr-AuNFs were recovered by centrifugation and then resuspended in pure water. N-AuNFs pH = 2 and Tr-AuNFs pH = 2 represent that N-AuNFs and Tr-AuNFs dissolve in a strongly acidic solution (pH = 2); N-AuNFs pH = 2+ and Tr-AuNFs pH = 2+ represent that the aggregated N-AuNFs and Tr-AuNFs were recovered by centrifugation and then resuspended in pure water.

To demonstrate the enhanced stability the N-AuNFs compared to the Tr-AuNFs, the resistance of both the AuNFs to various matrix interferences, including pH value and the ionic strength, were evaluated. Specifically, 8 fmol of Tr-AuNFs or N-AuNFs was added to 1 mL samples of water with different pH values or concentrations of NaCl, and the hydrodynamic size and LSPR spectra of the AuNFs were recorded after 5 min. The results show that both the Tr-AuNFs and N-AuNFs were stable in solutions with pH values of 6 to 14, and unstable in solutions with pH ≤ 5, in which they exhibited increased size (Fig. 1A) and decreased LSPR signal (Fig. 1B). As shown in Fig. 1C and D, NaCl concentrations of 2.5% or higher induced the aggregation of the Tr-AuNFs, resulting in a transparent solution with increased hydrodynamic size and decreased LSPR signal, while the N-AuNFs remained stable in 5% NaCl solution. The real photos of both the Tr-AuNFs and N-AuNFs treated with different concentrations of NaCl were displayed in Fig. S2 (ESI†). Moreover, unlike the Tr-AuNFs, the N-AuNF aggregates formed at a strongly acidic solution (pH = 2) and high NaCl concentration (20%, wt%) could be recovered by resuspension in pure water (Fig. 1E–H and S3†). Therefore, it was concluded that the Tr-AuNFs irreversibly aggregated in a strongly acidic solution and high-ionic strength solution due to the destruction of the hydration layer and the detachment of the citrate ligands, while the N-AuNFs exhibited higher stability and anti-inference ability because their strong Au–S bonds and the impermeability of the hydrophobic layer by water-soluble compounds prevented the detachment of the ligands.

### 3.2. Preparation and characterization of the AuNF probes

The AuNF probes including Tr-AuNF-Abs and N-AuNF-Abs were prepared by electrostatic adsorption and covalent coupling methods, respectively. Unpurified anti-ZEN ascites were directly immobilized onto the surface of the Tr-AuNFs according to the method described in our previous report.^17,31,32^ To obtain the highest binding affinity between Tr-AuNF-Abs and ZEN, the coupling parameters, including the pH value of the Tr-AuNF solution and the dosage of ascites, were optimized. Tr-AuNF-Abs obtained using different coupling conditions was used for strips with a BSA–ZEN conjugate as test line, and the optical density value at the test line (OD_T_) was used to evaluate the coupling efficiency and bioactivity of Tr-AuNF-Abs. The results shown in Fig. S4A† indicated that the optimized pH value for the preparation of Tr-AuNF-Abs was 7.9, which could be achieved by adding 1 μL of 0.2 M K_2_CO_3_ to 1 mL of Tr-AuNF solution (20 pM). Notably, the electrostatic adsorption coupling process was highly sensitive to change in the pH value, which would seriously decrease the reproducibility of the resulting Tr-AuNF-Abs. The optimal dosage of ascites for the preparation of Tr-AuNF-Abs was demonstrated to be 63 μg for 1 pmol Tr-AuNFs (Fig. S4B†). N-AuNF-Abs was prepared by coupling the amino group of anti-ZEN mAbs to the carboxyl group of N-AuNF *via* the carbodiimide method. To obtain the highest affinity of N-AuNF-Abs toward ZEN, the coupling parameters, including the pH value of the N-AuNF solution, the dosage of EDC, and the dosage of ascites were optimized, and the affinity of the resulting probes were evaluated *via* their performance in the strips. The results shown in Fig. S5A† demonstrate that the optimized pH value for the preparation of N-AuNF-Abs was 6.0. Compared to the preparation of Tr-AuNF-Abs, the covalent coupling process was much less sensitive to changes in the pH value, and thus would enable highly robust preparation of N-AuNF-Abs. Three or two portions of EDC solution were added regularly during the ascite immobilization process and BSA blocking process; each portion contained 5 μg EDC per 1 mL of N-AuNF solution (20 pM), according to the results shown in Fig. S5B.† The optimized dosage of ascites for the preparation of N-AuNF-Abs was demonstrated to be 125 μg for 1 pmol N-AuNFs (Fig. S5C†), which was higher than the dosage used in the preparation of Tr-AuNF-Abs due to the relatively lower coupling efficiency. The average *D*_H_ values of the resultant Tr-AuNF-Abs and N-AuNF-Abs were slightly higher than those of the Tr-AuNFs and N-AuNFs, respectively (Fig. S1C†). Slight red shifts in the SPR peaks of Tr-AuNF-Abs and N-AuNF-Abs were observed after immobilization of the antibody (Fig. S1D†).

**Fig. 2:**
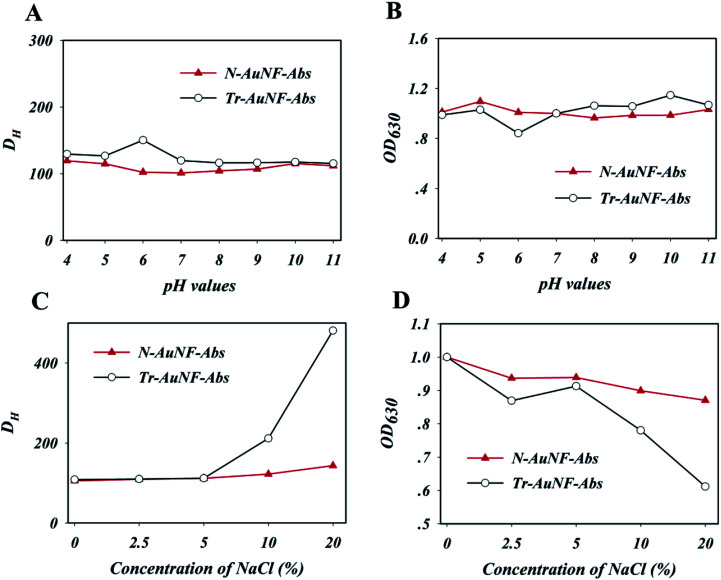
The effects of the pH values and NaCl concentrations on the hydration diameters (A and C) and optical intensity (OD_630_ value, B and D) of the N-AuNF-Abs and Tr-AuNF-Abs. OD_630_ values were normalized by dividing them by the OD_630_ value at pH = 7.0 or 0 wt% NaCl.

The resistance of the resulting Tr-AuNF-Abs and N-AuNF-Abs complexes against different pH values and ionic strengths (simulated by the concentration of NaCl) were evaluated. Specifically, to investigate the stability of the AuNF probes, 5 fmol of Tr-AuNF-Abs or N-AuNF-Abs was added to 1 mL of 0.01 M PB with different pH values or NaCl contents, and the hydrodynamic sizes and LSPR spectra of the AuNFs were recorded after 5 min. As demonstrated in Fig. 2A and B, both N-AuNF-Abs and Tr-AuNF-Abs showed high colloidal stability in the presence of a wide range pH values (4–11). The results in Fig. 2C demonstrate that Tr-AuNF-Abs in solutions with a NaCl content greater than 5 wt% exhibited a larger *D*_H_ and lower SPR signal than Tr-AuNF-Abs in a 0.01 M PB buffer (0 wt%), indicating the aggregation of Tr-AuNF-Abs; whereas the N-AuNF-Abs showed a negligible change in *D*_H_ and SPR signal even NaCl concentration up to 20 wt% (Fig. 2D), indicating that N-AuNF-Abs has a higher resistance against high ionic strength. Notably, compared with the corresponding AuNFs, both the AuNF probes showed higher resistance against high ionic strength or acidic conditions, which may have been due to the shielding effect of the proteins on their surfaces.

### 3.3. Development of AuNF probe-based ZEN-LFA

The two AuNF probes were then used to produce ZEN-LFA strips. The density of ZEN–BSA on the test line and the density of the AuNF probes on the conjugated pad were first optimized *via* an orthogonal experiment, in which the inhibition rate of a ZEN-spiked PB sample (5 ng mL^−1^) was used as the evaluation criterion. The inhibition rate was calculated as (1 − *B*/*B*_0_) × 100, where *B*_0_ and *B* represent the ratio of the OD values of the T line and C line (T/C) obtained from detecting the negative sample and a ZEN-positive sample, respectively. As shown in Table S1,† for the N-AuNF-Abs-based test strip, the optimized densities were 1.48 μg cm^−1^ ZEN–BSA and 1 fmol cm^−1^ N-AuNF-Abs, while for the Tr-AuNF-Abs based test strip, 0.37 μg cm^−1^ ZEN–BSA and 3 fmol cm^−1^ Tr-AuNF-Abs were optimum. Moreover, 15 min was selected as the reaction time for further experiments based on the results of immunological kinetics analysis (Fig. S6†). A high methanol content was necessary to achieve a high extraction recovery from the ZEN-polluted samples due to the strong hydrophobicity of ZEN, however, the presence of methanol in the sample solution can influence the antigen–antibody interactions. Thus, 0.01 M PB buffer solutions (pH = 7.0) with different methanol contents (0–40%) were tested with the two ZEN-LFA strips to evaluate the effect of methanol content. As shown in Fig. S7,† increasing the methanol content had an obvious effect on the signals (*i.e.*, OD_T_, and T/C values) of both strips. Thus, 5% was chosen as the optimum methanol content for subsequent experiments, as this concentration had little effect on the two ZEN-LFA strips.

**Fig. 3:**
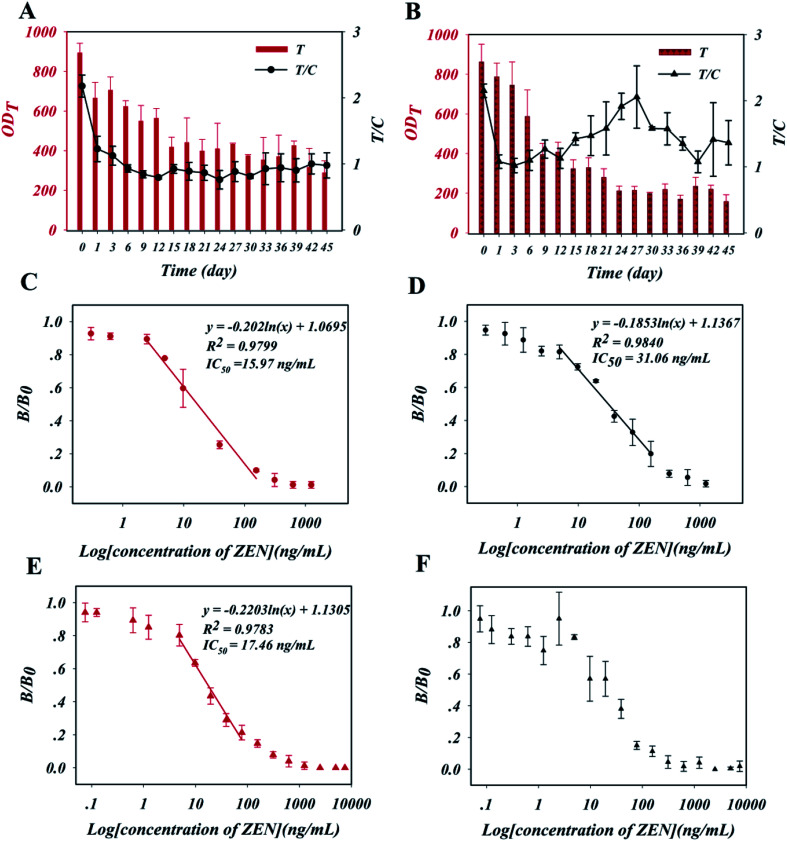
Changes in the signals (OD_T_, and T/C values) of the (A) N-AuNF-Abs and (B) Tr-AuNF-Abs based ZEN-LFA strips in response to negative samples (0.01 M PB buffer, pH = 7.0) during a 45 day aging period at 60 °C. Calibration curves of the (C) N-AuNF-Abs- and (D) Tr-AuNF-Abs-based ZEN-LFA strips for detecting ZEN-spiked PB (0.01 M, pH = 7.0) samples. Calibration curves of the (E) N-AuNF-Abs and (F) Tr-AuNF-Abs based ZEN-LFA strips for detecting ZEN-spiked DDGS extract (5% methanol) samples.

The stability of the as-prepared ZEN-LFA strips during long-term storage was explored using accelerated aging studies. Specifically, the strips were aged at 60 °C over a period of 45 days. Negative samples (0.01 M PB buffer, pH = 7.0) were added to the test strips regularly, and the OD_T_ and OD_C_ values were recorded. As shown in Fig. 3A, the resulting OD_T_ and OD_C_ values of the N-AuNF-Abs-based test trips decreased over the first six days of accelerated storage, then exhibited a plateau from day 7 to day 45 with a stabilized T/C value. In contrast, the OD_T_ and OD_C_ values of the Tr-AuNF-Abs-based test trips remained constant from day 1 to day 12 with a stable T/C value (Fig. 3B). Using the Arrhenius equation,^42,43^ it was preliminarily estimated that the shelf lives of the N-AuNF-Abs based test strips and Tr-AuNF-Abs based test strips at ambient temperature would be approximately 5 years and 1 year. The N-AuNF-Abs and Tr-AuNF-Abs based test strips were aged at 60 °C for 6 days and 24 h, respectively, after preparation, and then stored in a drying tower at ambient temperature for further usage.

The analytical performance of the two ZEN-LFA strips in the detection of ZEN-spiked PB buffer (0.01 M, pH = 7.0, 5% methanol) samples was then evaluated. Calibration curves (Fig. 3C and D) were obtained by plotting the resulting *B*/*B*_0_ values against the logarithm of the ZEN concentrations (0–1.25 μg mL^−1^), where *B* and *B*_0_ were T/C values obtained from detecting positive samples and negative samples, respectively. Then corresponding linear detection ranges, along with their regression equations and detection sensitivities (half-maximum inhibitory concentration, IC_50_), are also shown in Fig. 3C and D. N-LFA showed sensitive detection, with an IC_50_ of 15.97 ng mL^−1^ and a limit of detection (LOD, IC_10_) of 2.31 ng mL^−1^. This IC_50_ value was approximately half that of Tr-LFA (IC_50_ = 31.06 ng mL^−1^, LOD = 3.59 ng mL^−1^). Samples spiked with high concentrations (800 ng mL^−1^) of other mycotoxins (fumonisin B_1_, aflatoxin B_1_, aflatoxin B_2_, deoxynivalenol, aflatoxin G_1_, citrinin, and ochratoxin A) were used to evaluate the specificity of the two test strips. The results in Fig. S8† show that both ZEN-LFA strips could specifically detect ZEN.

### 3.4. Validation of the ZEN-LFA strips for ZEN detection in DDGS

The two ZEN-LFA strips were then used to detect ZEN in a DDGS sample. The primary DDGS extract solution was prepared by extracting a 1 g DDGS sample with 5 mL of the extraction solvent (40% v/v 0.01 M PB buffer, 60% methanol, pH = 7.0) for 30 min. The solution was then diluted 12-fold with 0.01 M PB buffer (pH = 7.0). Calibration curves (Fig. 3E and F) of the two ZEN-LFA strips for the detection of ZEN were prepared using spiked DDGS extract solution samples prepared from ZEN-negative DDGS samples. The N-LFA strips exhibited nearly the same sensitivity in the DDGS samples as in the PB buffer, with an IC_50_ of 17.46 ng mL^−1^ and LOD of 2.84 ng mL^−1^, indicating their high anti-interference ability (Fig. 3E). In contrast, the tests of ZEN-spiked DDGS extraction solutions using Tr-LFA strips yielded less robust data with much greater standard deviations; the IC_50_ could not be determined from the obtained calibration curve, which contained several discontinuities (Fig. 3F). Intra-day and inter-day assays were then conducted to evaluate the accuracy and reproducibility of the N-LFA strips using ZEN-spiked DDGS extract solutions with different concentrations (4.9, 19.5, and 78 ng mL^−1^). Intra-day assays were carried out within 1 day with five replicates at each spiking level, whereas inter-day assays were conducted on three consecutive days with five replicates at each spiking level. The average recoveries for the intra-day assays ranged from 98.5% to 124.3% with coefficients of variation (CVs) ranging from 2.8% to 14.5%, while the average inter-day recoveries ranged from 93.0% to 125.9% with CVs ranging from 7.0% to 21.9% (Table 1). The above results demonstrated that the N-AuNF-Abs-based strip was superior to the Tr-AuNF-Abs-based strip for the detection of ZEN in DDGS, with higher detection sensitivity, greater robustness, higher reproducibility, and better accuracy.

**Table 1:** Precision and accuracy evaluation of the N-AuNF-Abs-based strips with ZEN-spiked DDGS extract (5% methanol)

Spiked concentration (ng mL^−1^)	Intra-assay			Inter-assay^a^		
	Recovered concentration^b^ (ng mL^−1^)	Recovery (%)	CV (%)	Recovered concentration (ng mL^−1^)	Recovery (%)	CV (%)
4.9	4.8	98.5	7.5	4.6	93.0	16.9
19.5	27.6	111.9	14.5	26.4	125.9	21.9
78.0	97.0	124.3	2.8	120.5	120.5	7.0

a Assay was completed every 1 day for 3 days continuously.

b Mean value of five replicates at each spiked concentration.

The mechanism by which the matrix interfered with analytical performance of the Tr-LFA strips was then explored. In theory, the negative impact of the sample matrix on the ZEN-LFA strips may have been due to its influence on antigen–antibody interactions or decreased stability of the AuNF probes. First, the effects of some common sources of matrix interference, including the pH and ionic strength, on the two ZEN-LFA strips were evaluated. Specifically, negative samples (0.01 M PB buffer) containing different matrix interferents were tested using the two ZEN-LFA strips. The results in Fig. S9A and B† demonstrate that the two ZEN-LFA strips showed similar changes in their OD_T_ and OD_C_ responses with changing pH values (4–11), which may account for the influence of the pH value on the antigen–antibody interaction on the T line and C line. As shown in Fig. S9C and D,† the presence of 0–2.5 wt% NaCl in the samples (0.01 M PB, pH = 7.0) had little effect on either of the ZEN-LFA strips. Thus, it was concluded that the effect of common matrix interferents, including the pH value and ionic strength, could account for the changes in antigen–antibody interactions, and no obvious differences in the responses of the two ZEN-LFA strips to these interferents were observed.

**Fig. 4:**
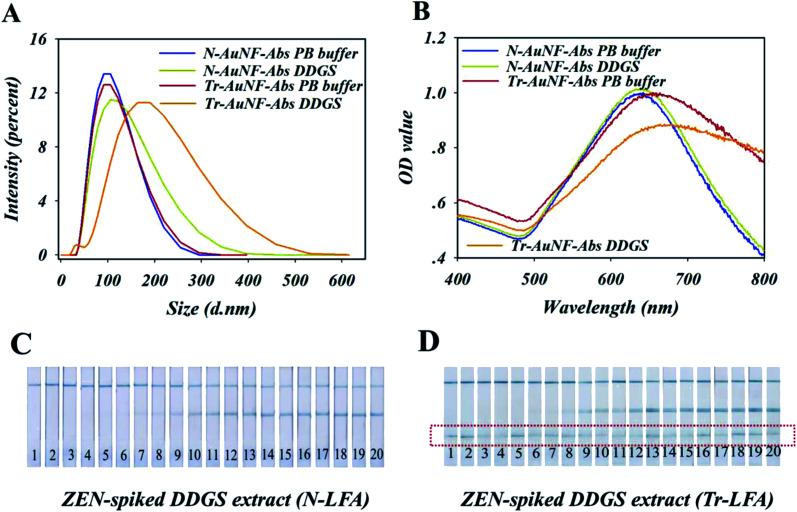
(A) DLS analysis and (B) UV-Vis spectra of N-AuNF-Abs and Tr-AuNF-Abs suspended in DDGS extractions (5% methanol) and PB buffer (pH = 7.0), recorded 5 min after the addition of the AuNFs. Images of test strip responses to different samples, (C) N-AuNF-Abs and (D) Tr-AuNF-Abs based strips with ZEN-spiked DDGS extract (5% methanol). Samples 1–20 of each group were spiked with different concentrations of ZEN from 0 to 7.5 μg mL^−1^ (the corresponding concentrations of 1–20 are 7.5 μg mL^−1^, 5.0 μg mL^−1^, 2.5 μg mL^−1^, 1.25 μg mL^−1^, 625 ng mL^−1^, 313 ng mL^−1^, 156 ng mL^−1^, 78 ng mL^−1^, 39 ng mL^−1^, 20 ng mL^−1^, 9.8 ng mL^−1^, 4.9 ng mL^−1^, 2.45 ng mL^−1^, 1.225 ng mL^−1^, 0.613 ng mL^−1^, 0.3 ng mL^−1^, 0.15 ng mL^−1^, 0.075 ng mL^−1^, 0.038 ng mL^−1^, and 0, respectively).

Therefore, the high robustness of N-LFA strips for the detection of ZEN in DDGS may have been the result of the high stability and anti-interference ability of N-AuNF-Abs, which were provided by the amphiphilic ligand and the covalent coupling of the antibodies, while the lower stability of Tr-AuNF-Abs could be accounted for by the decreased colloidal stability or the detachment of the antibodies. To test these assumptions, an ELISA-based experiment to quantify the detachment of the antibodies was conducted. Specifically, three different solutions (0.01 M PBS containing 20%, 40%, or 60% methanol, pH = 7.4) were used to extract ZEN-negative DDGS, and then three different DDGS extractions (5% methanol) containing different matrix interferents were obtained by dilution with PBS buffer. The two AuNF probes (12.5 fmol) were separately incubated with 350 μL of each of the three DDGS extractions or PBS buffer for 30 min, followed by a centrifugation process. The concentrations of anti-ZEN antibodies in the eight resulting supernatants were detected using an established conventional ELISA technique, in which BSA–ZEN conjugate was used as the capture probe and horseradish peroxidase (HRP)-IgG conjugate was used as the reporter (Fig. S10†). The results showed the negligible content of antibodies was found in the supernatant, indicating that the detachment of the antibody was not the reason for the low stability of Tr-AuNF-Abs in DDGS. However, Tr-AuNF-Abs exhibited the remarkably increased *D*_H_ and decreased SPR signal in the DDGS extract, while the *D*_H_ and SPR signal of N-AuNF-Abs were almost the same in both PBS and DDGS extract (Fig. 4A and B). In addition, compared with N-AuNF-Abs, the significantly clogging of the AuNF probes has occurred on the Tr-AuNF-Abs based strips when the ZEN-spiked DDGS samples were used (Fig. 4C, D and S11†). Therefore, it can be inferred that aggregation of Tr-AuNF-Abs induced by matrix interference was the primary reason for the low robustness of the Tr-AuNF-Abs-based strips. We speculated that possible mechanism for interferent-induced aggregation of the Tr-AuNF-Abs could involve the replacement of the BSA on the surfaces of the AuNFs by interferents in the DDGS. For instance, BSA could be replaced by liposoluble substances or substances with polyvalent bonding ability towards AuNFs (*e.g.*, proteins with multiple thiol groups available), while for N-AuNF-Abs, such replacement could be avoided.

## 4. Conclusions

Herein, a novel AuNF probe (N-AuNF-Abs) with enhanced stability was prepared by immobilizing antibodies onto AuNFs capped with amphiphilic ligands terminated by thiol and carboxyl groups *via* covalent coupling. A traditional AuNF probe (Tr-AuNF-Abs) was also prepared by modifying the antibodies onto citrate-capped AuNFs *via* electrostatic adsorption and used as a comparison. The Tr-AuNF-Abs probe showed interference-induced aggregation, as a result, the ZEN-LFA based on this probe was not robust enough for the analysis of DDGS samples. The as-prepared N-AuNF-Abs probe showed higher stability and anti-interference ability, with no interference-induced aggregation. The N-AuNF-Abs based LFA showed high robustness in the detection of ZEN in DDGS samples. Under optimal conditions, the proposed strips exhibited nearly the same sensitivity, with an IC_50_ of 17.46 ng mL^−1^ and LOD of 2.84 ng mL^−1^, for detection in DDGS samples and in PB buffer. The average recoveries for the detection of ZEN-spiked DDGS samples ranged from 90.0% to 130.0% with a CV of less than 22%, indicating the high accuracy and excellent reproducibility of the proposed ZEN-LFA strips. In conclusion, the N-AuNF-Abs-based LFA achieved highly sensitive and robust detection of ZEN in DDGS samples, and also showed promising potential for the determination of other pollutants in complex sample matrices.

## Conflicts of interest

There are no conflicts to declare.

## Supplementary Material

RA-009-C9RA06690J-s001
