# Supplementary material for: An amphiphilic-ligand-modified gold nanoflower probe for enhancing the stability of lateral flow immunoassays in dried distillers grains
Source: RSC Adv. 2019 Nov 11;9(63):36670–9. doi: 10.1039/c9ra06690j (PMC9075177; doi:10.1039/c9ra06690j)
Supplement: RA-009-C9RA06690J-s001 [file RA-009-C9RA06690J-s001.pdf]

Electronic Supplementary Material (ESI) for RSC Advances.
This journal is © The Royal Society of Chemistry 2019

Supporting information for

An amphiphilic-ligand-modified gold nanoflower probe for enhancing the stability of lateral flow immunoassays in dried distillers' grains

Tongtong Ma[a,b], Hong Duan[a,b], Wenjing Zhang[a,b], Yanna Shao[a,b], Liangwen Hao[a,b], Yuankui Leng[*a,c], Xiaolin Huang[a,c], Yonghua Xiong[*a,b]

[a] State Key Laboratory of Food Science and Technology, Nanchang University, Nanchang 330047, P. R. China;

[b] Jiangxi-OAI Joint Research Institute, Nanchang University, Nanchang 330047, P. R. China;

[c] School of food science and technology, Nanchang University, Nanchang 330047, P. R. China;

*Correspondence to:

Dr. Yuankui Leng and Dr. Yonghua Xiong

State Key Laboratory of Food Science and Technology, and Jiangxi-OAI Joint Research Institute, Nanchang University, Nanchang 330047, P.R. China

E-mail: yuankuilengxq@163.com ; yhxiongchen@163.com

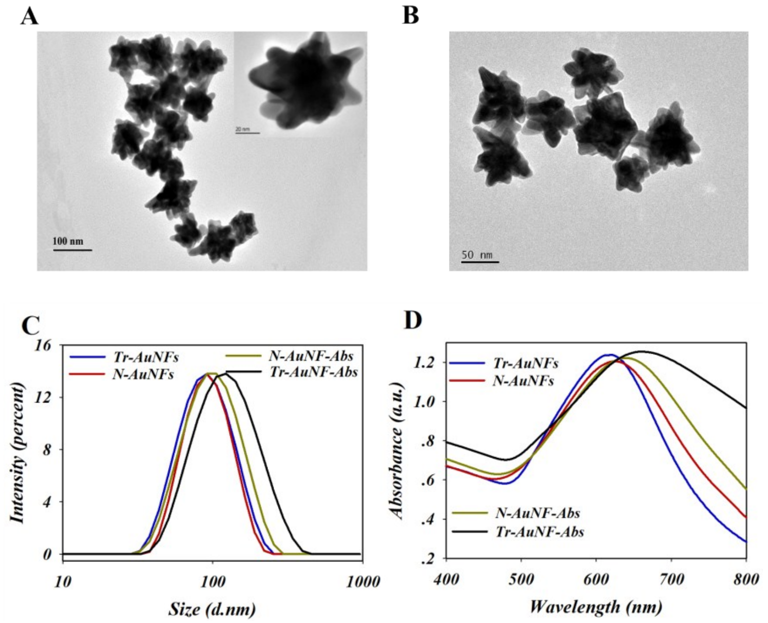

Fig. S1. Characterization of the 80 nm Tr-AuNFs, N-AuNFs, N-AuNF-Abs, and Tr-AuNF-Abs: (A) TEM images of the 80 nm Tr-AuNFs, (B) TEM image of the 80 nm N-AuNFs. (C) DLS analysis and (D) UV-Vis spectra of the 80 nm Tr-AuNFs, N-AuNFs, N-AuNF-Abs, and Tr-AuNF-Abs.

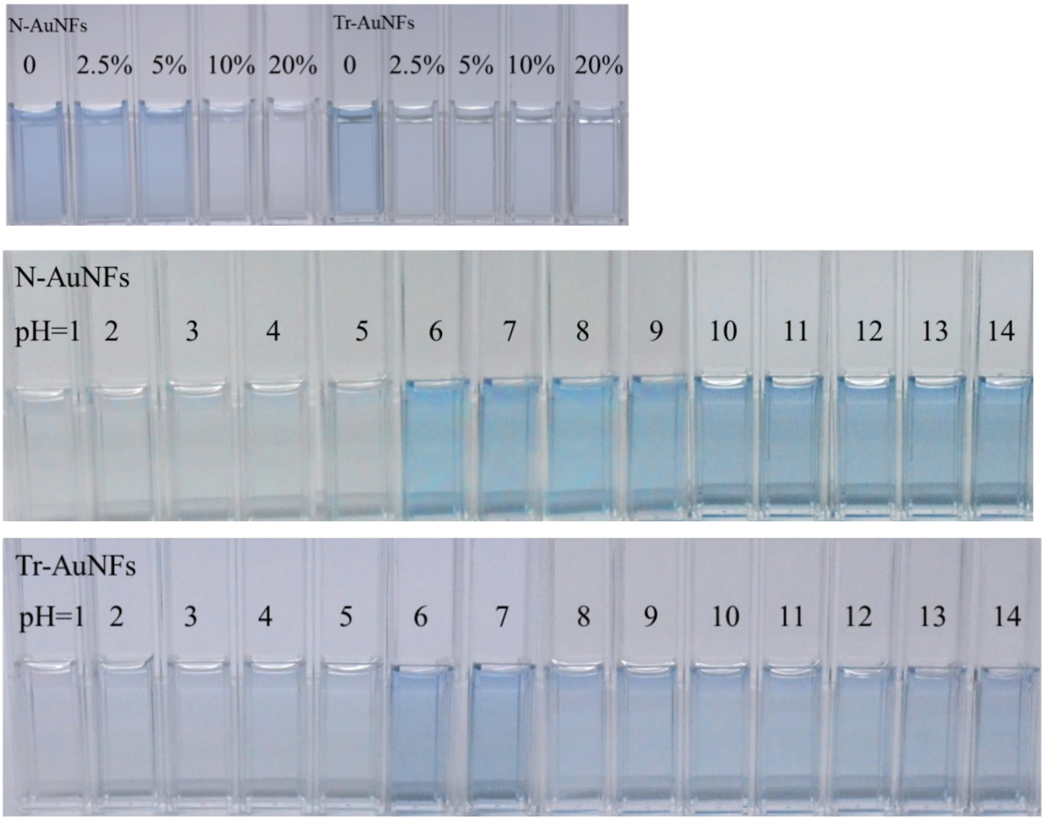

Fig. S2. Real photos of both the Tr-AuNFs and N-AuNFs treated with different concentrations of NaCl (0-20 wt.%) and different pH values (1-14)

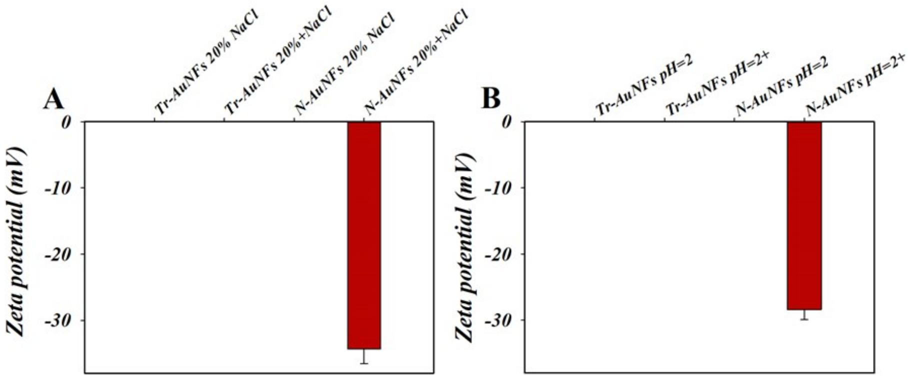

Fig. S3. (A) Zeta potentials of N-AuNFs and Tr-AuNF treated with 20% of NaCl solution (wt.%); (B) Zeta potential of N-AuNFs and Tr-AuNFs treated with a strongly acidic solution (pH = 2); N-AuNFs 20% NaCl and Tr-AuNFs 20% NaCl represent that N-AuNFs and Tr-AuNFs dissolve in 20% of NaCl solution (wt.%); N-AuNFs 20%+NaCl and Tr-AuNFs 20%+NaCl represent that the aggregated N-AuNFs and aggregated Tr-AuNFs recovered by centrifugation were resuspended in pure water; N-AuNFs pH=2 and Tr-AuNFs pH=2 represent that N-AuNFs and Tr-AuNFs dissolve in a strongly acidic solution (pH = 2); N-AuNFs pH=2+ and Tr-AuNFs pH=2+ represent that the aggregated N-AuNFs and Tr-AuNFs recovered by centrifugation were resuspended in pure water.

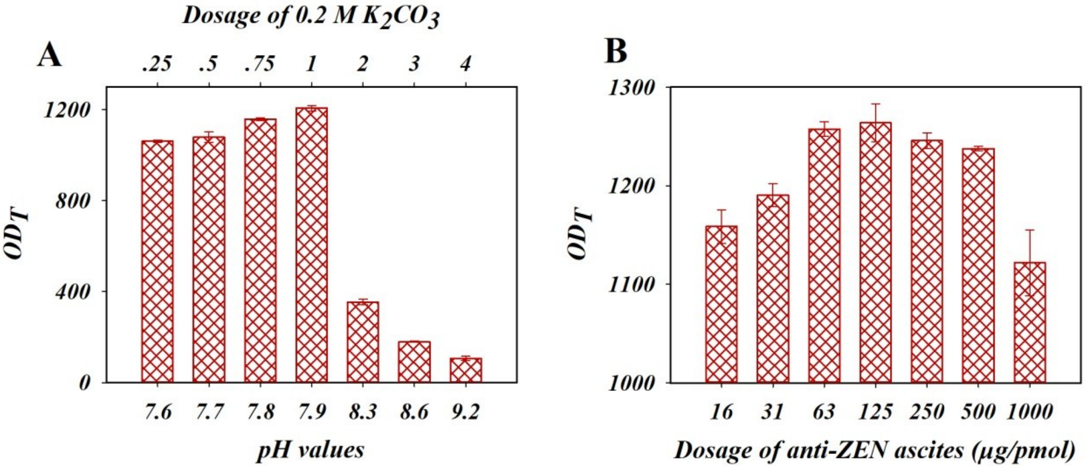

Fig. S4. Parameter optimization for the preparation of Tr-AuNF-Abs. OD_T values of test strips based on Tr-AuNF-Abs prepared using different (A) pH values, which were adjusted by adding 0.2 M K_2CO_3 to the Tr-AuNF solution (20 pM), and (B) dosages of anti-ZEN ascites.

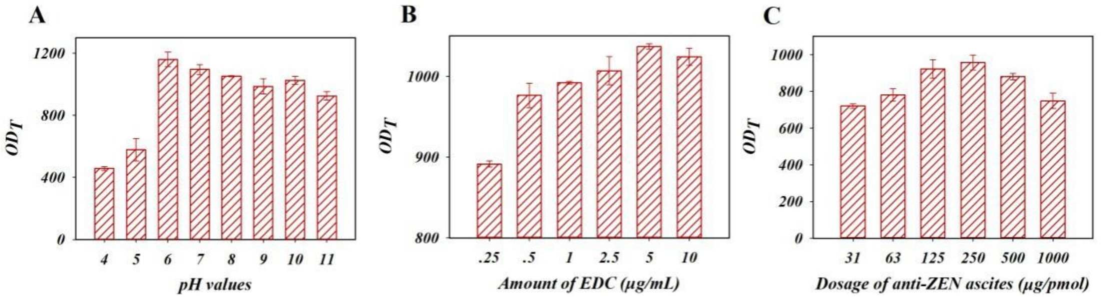

Fig. S5. Parameter optimization for the preparation of N-AuNF-Abs. OD_T values of test strips based on Tr-AuNF-Abs prepared using different (A) pH values, (B) amounts of EDC, and (C) dosages of anti-ZEN ascites.

Table S1 Optimization of the parameters of the (A) N-AuNF-Abs- and (B) Tr-AuNF-Abs-based ZEN-LFA strips.

A

No.	N-AuNF-Abs on conjugate pad (fmol/cm)	ZEN-BSA on T line (μg/cm)	Negative Sample		5 ng/ml ZEN Sample
			OD_T	OD_C	Competitive inhibition ratio
1	1	0.37	443±24	325±21	41.6
2	1	0.74	536±99	137±23	52.5
3*	1	1.48	618±80	108±8	63.7
4	1.5	0.37	578±51	430±28	33.5
5	1.5	0.74	658±51	173±25	42.7
6	1.5	1.48	831±94	149±21	48.2
7	2	0.37	653±119	520±77	28.5
8	2	0.74	690±94	193±38	42.7
9	2	1.48	1016±28	180±36	51.7

B

No.	Tr-AuNF-Abs on conjugate pad (fmol/cm)	ZEN-BSA on T line (μg/cm)	Negative Sample		5 ng/ml ZEN Sample
			OD_T	OD_C	Competitive inhibition ratio
1*	3	0.37	547±39	248±25	55.6
2	3	0.74	566±29	136±34	48.9
3	3	1.48	752±31	59±7	39
4	3.5	0.37	541±16	299±32	30.5
5	3..5	0.74	651±58	123±10	27.1
6	3.5	1.48	738±13	59±21	53.4
7	4	0.37	580±14	314±38	33.2
8	4	0.74	674±34	154±27	27.8
9	4	1.48	844±4	756±13	17

*: represents the optimum parameters for the preparation of ZEN-LFA strip

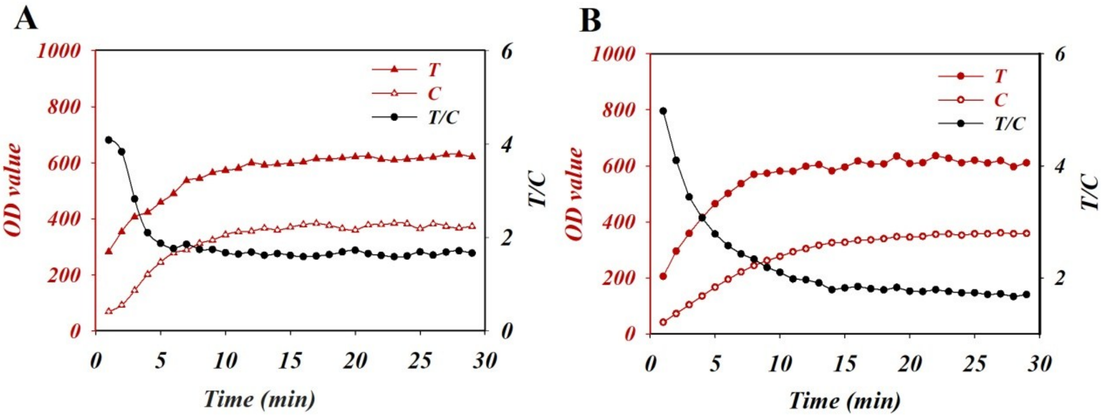

Fig. S6. Immunoreaction dynamic curves for the (A) N-AuNF-Abs- and (B) Tr-AuNF-Abs-based ZEN-LFA strips.

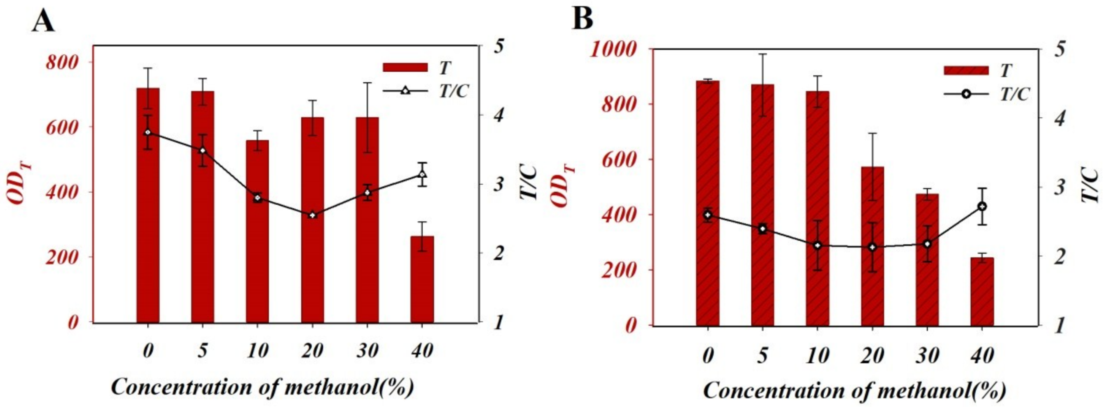

Fig. S7. Effects of the methanol content on the detection performance of the (A) N-AuNF-Abs- and (B) Tr-AuNF-Abs-based ZEN-LFA strips.

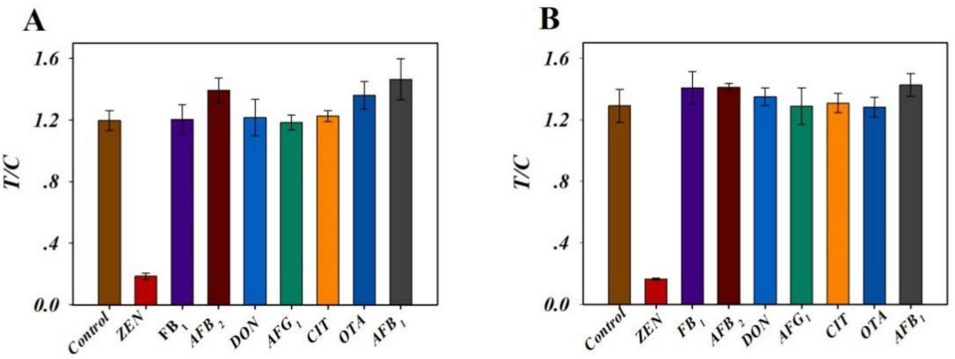

Fig. S8. (A) Specificity evaluation of the N-AuNF-Abs based ZEN-LFA strips, in which PB buffer (0.01 M, pH=7, 5% methanol) spiked with various mycotoxins was tested. Samples spiked with 800 ng/mL FB_1, AFB_2, DON, AFG_1, CIT, OTA, and AFB_1 and 80 ng/mL of ZEN were used as the positive control, while blank PB buffer was used as the negative control (B) Specificity evaluation of Tr-AuNF-Abs based ZEN-LFA strips, in which PB buffer (0.01 M, pH=7, 5% methanol) spiked with various mycotoxins was tested. Samples spiked with 1600 ng/mL of FB_1, AFB_2, DON, AFG_1, CIT, OTA, and AFB_1 and 160 ng/mL of ZEN were used as the positive control, while blank PB buffer was used as the negative control.

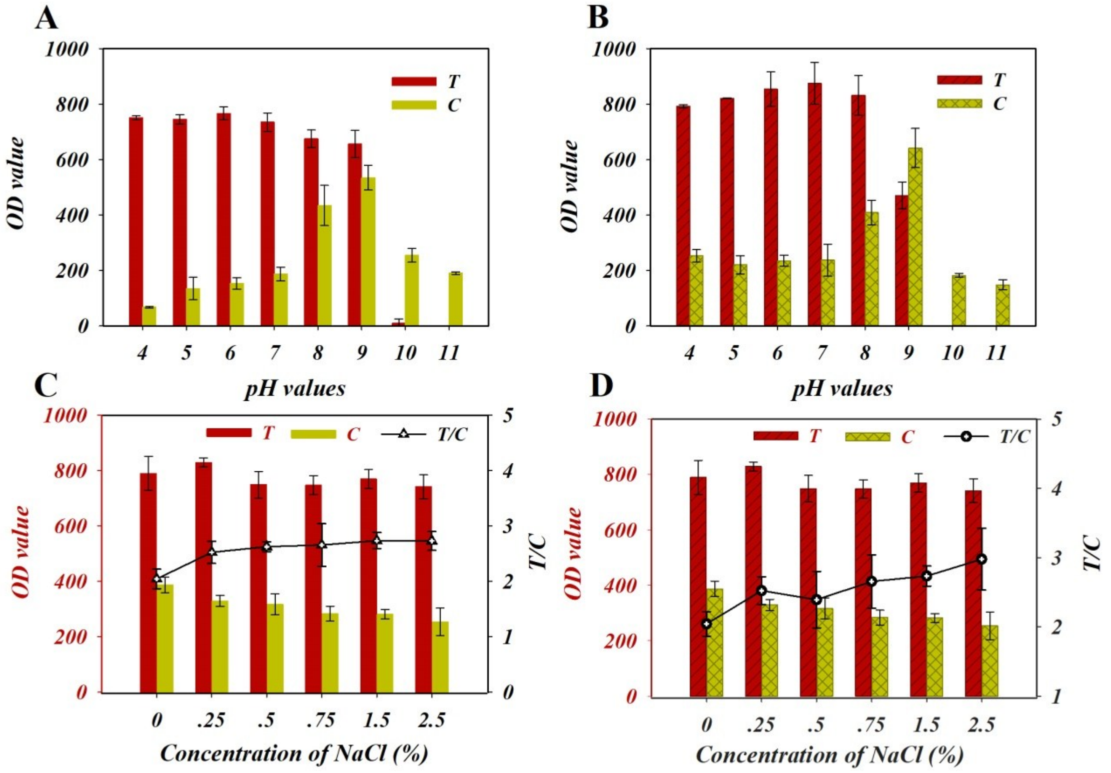

Fig. S9. Effects of some common matrix interferents on the N-AuNF-Abs based strip (A, C) and Tr-AuNF-Abs based strip (B, D); different pH values (A, B) and ionic strengths (C, D) were evaluated.

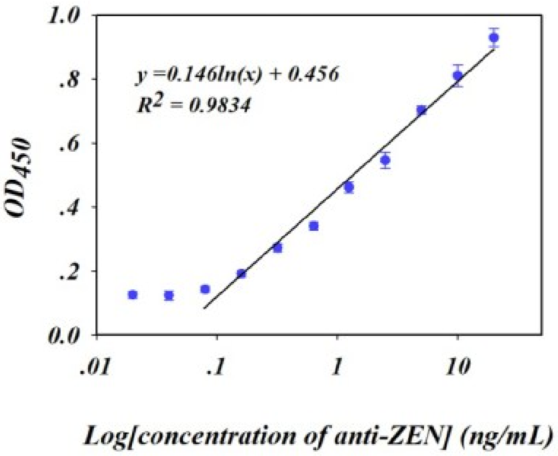

Fig. S10. Indirectly ELISA for anti-ZEN antibody determination

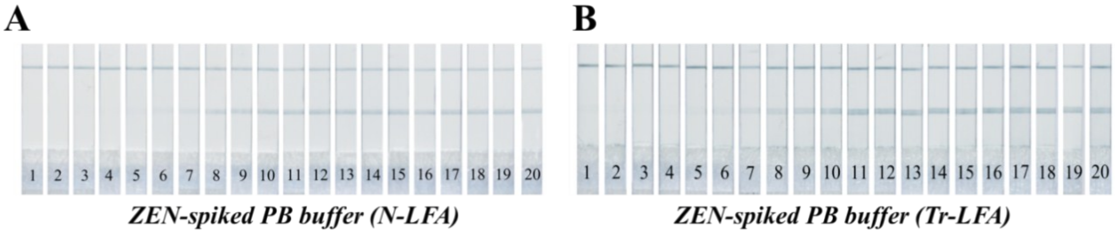

Fig. S11. Images of test strip responses to PB buffer. (A) N-AuNF-Abs- and (B) Tr-AuNF-Abs-based strips with ZEN-spiked PB buffer. Samples 1-20 of each group were spiked with different concentrations of ZEN from 0 to 7.5 μg/mL (The corresponding concentrations of 1-20 are 7.5 μg/mL, 5.0 μg/mL, 2.5 μg/mL, 1.25 μg/mL, 625 ng/mL, 313 ng/mL, 156 ng/mL, 78 ng/mL, 39 ng/mL, 20 ng/mL, 9.8 ng/mL, 4.9 ng/mL, 2.45 ng/mL, 1.225 ng/mL, 0.613 ng/mL, 0.3 ng/mL, 0.15 ng/mL, 0.075 ng/mL, 0.038 ng/mL, and 0, respectively).
